# Integrative single-cell eQTL and multi-omics analyses reveal AIM1 and ANXA1 as immune-related hub genes and potential therapeutic targets in head and neck cancer

**DOI:** 10.3389/fonc.2026.1912193

**Published:** 2026-08-06

**Authors:** Zhangwei Xue, Guohang Shen, Gongbiao Lin, Hanzhen Xue, Ruoyan Wang, Yue Zhou, Yang Chen, Kaiyong Wang, Yupei Dai

**Affiliations:** 1 Affiliated Hospital of Putian University, The Graduate School of Fujian Medical University, Putian, Fujian, China; 2 Department of Otolaryngology–Head and Neck Surgery, The First Affiliated Hospital of Fujian Medical University, Fuzhou, China; 3 North Sichuan Medical College, Nanchong, Sichuan, China; 4 Department of Clinical Medicine, Ningxia Medical University, Yinchuan, Ningxia Hui Autonomous Region, China; 5 School of Basic Medicine and Forensic Medicine, North Sichuan Medical College, Nanchong, Sichuan, China

**Keywords:** FAERS database, head and neck cancer, molecular dynamics simulation, sc-eQTL, single-cell analysis, spatial transcriptomics

## Abstract

**Introduction:**

Head and neck cancer (HNC) is characterized by substantial immune heterogeneity and limited availability of clinically actionable molecular targets. Here, we developed an integrative single-cell eQTL-driven multi-omics framework to identify immune cell-specific causal genes and prioritize drug-repurposing candidates for HNC.

**Methods:**

Single-cell eQTL data from the OneK1K cohort were integrated with European HNC GWAS summary statistics through two-sample Mendelian randomization, followed by transcriptomic differential expression analysis and weighted gene co-expression network analysis. Candidate targets were further evaluated using diagnostic modeling, immune infiltration analysis, single-cell and spatial transcriptomics, Bayesian colocalization, Western blot validation, molecular docking, molecular dynamics simulations, and FAERS-based safety profiling.

**Results:**

We identified 494 immune cell-specific eGenes causally associated with HNC risk. Multi-layered target prioritization highlighted AIM1 and ANXA1 as protective immune-related genes, both of which were downregulated in HNC tissues and showed cell-type-preferential expression in T cells and monocytes, respectively. A dual-gene diagnostic model achieved strong discrimination performance with an AUC of 0.917. Colocalization analysis supported shared genetic signals between AIM1 or ANXA1 loci and HNC susceptibility, while Western blotting confirmed reduced AIM1 and ANXA1 protein expression in SCC-9 cells compared with normal HOK cells. Drug screening and molecular docking identified topotecan as a candidate ligand for AIM1 and terbutaline as a candidate ligand for ANXA1. Subsequent molecular dynamics simulations demonstrated stable drug–target complexes with favorable binding free energies. FAERS analysis further characterized the adverse-event spectrum and potential safety considerations for both compounds.

**Discussion:**

Collectively, this study provides a single-cell genetic and pharmacological framework for defining immune-related causal targets in HNC and supports AIM1 and ANXA1 as promising biomarkers and therapeutic entry points for precision drug development.

## Graphical Abstract

**Figure:**
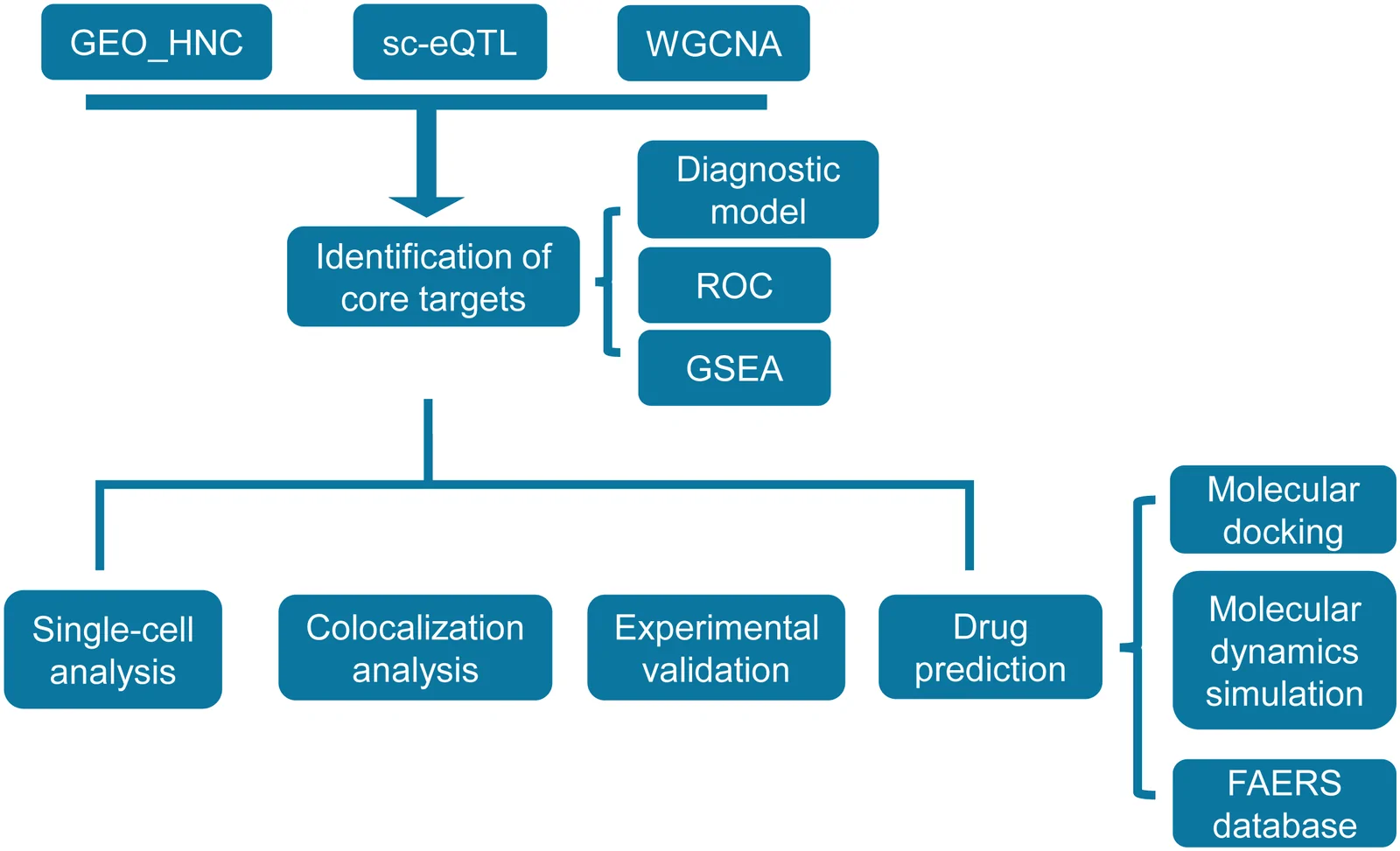

## 1 Introduction

Head and neck cancer (HNC) encompasses a heterogeneous group of malignancies arising from the mucosal epithelium of the head and neck region, ranking among the most prevalent and lethal cancers worldwide. The pathogenesis of HNC is multifactorial, involving genetic variants, immune microenvironment dysregulation, and aberrant signaling pathways and inflammatory responses (1–3). The European population represents one of the high-risk groups for HNC; however, clinically, there remains a lack of highly specific biomarkers for early detection and a scarcity of effective molecular targets for precision therapy. Most patients are diagnosed at advanced stages, and conventional interventions such as surgery, radiotherapy, chemotherapy, as well as existing targeted or immunotherapeutic strategies, often yield limited efficacy, resulting in suboptimal prognosis (4–6). Therefore, the identification of key molecular targets with both diagnostic and therapeutic potential, coupled with elucidation of their mechanistic roles in disease initiation and progression, represents a pivotal challenge in advancing both basic and translational research in HNC.

The aberrant remodeling of the tumor immune microenvironment (TIME) constitutes a pivotal regulatory mechanism underlying the initiation, invasion, and metastasis of head and neck cancer. The infiltration patterns and functional states of distinct immune cell subsets play decisive roles in tumor progression, while dysregulation of immune cell type-specific gene expression represents a fundamental molecular driver of immune microenvironment imbalance (7, 8). Expression quantitative trait loci (eQTL) offer a robust framework to elucidate the genetic regulation of gene expression, revealing causal effects of genetic variants on transcriptional activity. However, conventional bulk tissue-based eQTL analyses are limited by cellular heterogeneity, impeding precise characterization of gene regulatory networks across distinct immune cell subpopulations (9). Single-cell eQTL (sc-eQTL) approaches overcome these limitations, enabling high-resolution mapping of the associations between genetic variants and gene expression within specific immune cell subsets, thereby providing a novel avenue for accurately identifying disease-associated genes with immune cell type specificity (10, 11). Moreover, two-sample Mendelian randomization (MR), leveraging genetic variants as instrumental variables, effectively mitigates confounding and facilitates causal inference between exposures and disease outcomes, establishing a reliable analytical framework for selecting causal immune cell-specific genes in HNC from sc-eQTL data (12). Notably, current studies have yet to comprehensively investigate the spatiotemporal expression profiles (including single-cell level cellular distributions and tissue-level spatial localization), genetic colocalization patterns, and potential targeted therapeutics of core HNC targets, limiting their translational utility in precision diagnostics and targeted treatment strategies.

Building on this framework, the present study utilized sc-eQTL data from the OneK1K dataset, in conjunction with large-scale summary statistics from a HNC genome-wide association study (GWAS; ieu-b-4912), to perform two-sample MR analyses of 17 immune cell type-specific eGenes, aiming to identify causal immune-related genes associated with HNC susceptibility. Subsequently, differential gene expression analysis and WGCNA were integrated to pinpoint potential core targets through a multi-layered intersection strategy. The identified core targets were comprehensively validated across multiple dimensions, including diagnostic performance, functional characterization, modulation of immune cell infiltration, spatiotemporal expression patterns, and genetic colocalization. Furthermore, potential therapeutic compounds targeting these core genes were systematically screened and evaluated via molecular docking to assess binding affinity and interaction specificity. Collectively, this study establishes an sc-eQTL-guided framework for the systematic identification of immune-related causal genes in HNC, providing novel molecular biomarkers for precision immunodiagnosis and actionable targets for the development of targeted therapeutics.

## 2 Methods

### 2.1 Two-sample mendelian randomization analysis of immune cell type-specific eGenes in head and neck cancer

sc-eQTL data from the OneK1K dataset were employed as the exposure variables, while summary statistics from a European cohort of HNC GWAS (ieu-b-4912; 9,655,080 SNPs, 1,106 cases, 372,016 controls) were used as the outcome. Seventeen immune cell type-specific eGenes were evaluated using a two-sample Mendelian randomization (MR) framework. SNPs associated with the exposure were initially selected at a genome-wide significance threshold of P < 5 × 10^-8^, relaxed to P < 1 × 10^-5^ if insufficient instrumental variables were available. Linkage disequilibrium (LD) pruning was subsequently performed (r² < 0.001, window = 10 Mb) to remove highly correlated variants. Allele harmonization and standardization were conducted using the TwoSampleMR package, excluding palindromic and mismatched SNPs, and weak instruments were filtered using an F-statistic threshold > 10. For exposures with ≥2 independent instruments, the inverse variance weighted (IVW) method was applied as the primary causal estimator, supplemented with weighted median and MR-Egger regression. For exposures with only one instrument, the Wald ratio method was used. Sensitivity analyses included Cochran’s Q test for heterogeneity, MR-Egger intercept, MR-PRESSO global test, and leave-one-out analysis. Following false discovery rate (FDR) correction, eGenes significantly associated with HNC risk and exhibiting robust causal effects were retained (10, 13, 14).

### 2.2 Differential gene expression analysis and weighted gene co-expression network analysis in head and neck cancer

HNC transcriptomic datasets were retrieved from the GEO database, merged, and corrected for batch effects. Differential gene expression analysis was performed using the limma package, with a threshold of |log_2_ fold change| ≥ 0.5 to identify up- and downregulated genes. WGCNA was performed to identify HNC-associated gene co-expression patterns. Module–trait correlations were evaluated to prioritize phenotype-associated gene sets, which were subsequently integrated with differential expression and genetic evidence for candidate target identification (15, 16).

### 2.3 Identification of core target genes

Core target genes were identified through an integrative intersection analysis using the Venn package, combining differentially expressed genes, WGCNA module genes, and immune cell type-specific eGenes obtained from MR analyses. The screening procedure involved two steps: first, the intersection of upregulated differentially expressed genes, positively correlated WGCNA module genes, and risk-associated eGenes (OR>1) was obtained; second, the intersection of downregulated genes, negatively correlated WGCNA module genes, and protective eGenes (OR<1) was calculated. The union of these two intersections constituted the set of core HNC target genes, serving as the foundation for subsequent functional validation and potential clinical applications.

### 2.4 Construction and performance evaluation of diagnostic models

Diagnostic models for HNC were constructed based on the identified core genes. Receiver operating characteristic (ROC) curves and area under the curve (AUC) metrics were employed to evaluate the diagnostic performance of individual genes and the combined model. Nomograms were generated to visualize disease risk prediction, and calibration curves were used to assess the agreement between predicted probabilities and observed outcomes, providing a comprehensive evaluation of the model’s clinical applicability (17).

### 2.5 Functional characterization of core genes and immune infiltration analysis

Gene-gene expression correlations among core targets were analyzed using ggpubr and corrplot, with chromosomal localization determined via the circlize package. Protein–protein interaction (PPI) networks were constructed using the GeneMANIA database to elucidate inter-target relationships. Functional enrichment analyses were conducted with org.Hs.eg.db and clusterProfiler, employing the c2.cp.kegg.Hs.symbols.gmt gene set for gene set enrichment analysis (GSEA; P < 0.05) to investigate pathway-level associations. Single-sample GSEA (ssGSEA) was performed using the GSVA package to quantify the infiltration of 28 immune cell subsets across tumor and normal tissues, and ggplot2 was employed to visualize differential immune infiltration. Correlation analyses were further conducted to evaluate the relationship between core gene expression and immune infiltration scores (18, 19).

### 2.6 Single-cell RNA sequencing data analysis

Single-cell RNA sequencing (scRNA-seq) datasets of head and neck cancer (HNC) were obtained from the GEO database and processed using the Seurat package. Low-quality cells were excluded based on the following criteria: nFeature_RNA < 200 or > 5000, nCount_RNA < 1000, and mitochondrial gene expression > 5%. The data were then normalized using LogNormalize, and 2,000 highly variable genes (HVGs) were selected. Potential confounding effects from mitochondrial, ribosomal, and cell cycle-related genes were regressed out using the ScaleData function. Dimensionality reduction and clustering were performed using the UMAP algorithm, and cell-type annotation was conducted with the SingleR package to assess the cell type-specific expression patterns of core target genes (20, 21).

### 2.7 Spatial transcriptomics data analysis

Spatial transcriptomic datasets were processed using Seurat. UMI counts were normalized and standardized, and highly variable genes were identified via the SCTransform function. Principal component analysis (PCA) was performed using RunPCA (top 30 PCs), followed by unsupervised clustering with FindNeighbors and FindClusters. SpatialFeaturePlot was employed to visualize the spatial distribution of cell subpopulations and the tissue-level spatial expression patterns of core genes (22). Spatial transcriptomic analysis was performed using the publicly available GSE252265 dataset from the Gene Expression Omnibus (GEO) database. GSE252265 was derived from human head and neck squamous cell carcinoma (HNSCC) tissues and generated using the 10X Genomics Visium Spatial Gene Expression platform. The samples were collected in 2022 from Helsinki University Hospital, Finland. Tissue specimens were freshly frozen in OCT, sectioned into 10 μm-thick slices, and subsequently processed using Visium spatial transcriptomics arrays for spatial gene expression profiling. In this study, the dataset was utilized to characterize the spatial localization patterns of AIM1 and ANXA1 within the HNSCC tissue microenvironment.

### 2.8 Bayesian colocalization analysis of core target genes

To investigate the genetic causal association of core targets, Bayesian colocalization analysis was performed using the coloc package, integrating core gene eQTL data with HNC GWAS summary statistics. Five mutually exclusive genetic association hypotheses were evaluated, and posterior probabilities (PP) were calculated. A PP4 > 0.8 was considered strong evidence for shared causal variants between core gene eQTL loci and HNC-associated genetic variants (23).

### 2.9 Prediction of potential therapeutic compounds

Core target genes were used to query the DSigDB database to identify potential therapeutic compounds. The top ten ranked compounds were selected, excluding those with high toxicity, carcinogenicity, or teratogenicity. FDA-approved drugs were retained as candidate compounds to ensure clinical feasibility and safety.

### 2.10 Molecular docking of candidate drugs with core targets

To investigate the potential molecular interactions between drug repurposing-derived candidate compounds and immune-related core targets, molecular docking analysis was performed between the selected compounds and their corresponding target proteins. The three-dimensional structures of AIM1 and ANXA1 proteins were obtained from the Protein Data Bank (PDB) and AlphaFold Protein Structure Database, respectively. Before docking analysis, the protein structures were subjected to standard preprocessing procedures, including the removal of non-essential small molecules and water molecules, addition of missing hydrogen atoms, and structural optimization.

The three-dimensional structures of candidate drug molecules were retrieved from the PubChem database in SDF format and subsequently converted into docking-compatible formats. Prior to docking, ligand structures were subjected to energy minimization and structural optimization. Molecular docking analysis was performed using AutoDock, with the docking search region defined according to the potential binding sites of the target proteins. All other docking parameters were set according to the default workflow of the software. The binding capability between candidate compounds and target proteins was evaluated based on the predicted binding energy. A binding energy threshold of < −5 kcal/mol, which has been commonly applied as an initial screening criterion in previous virtual screening studies, was used only for prioritizing potential ligand–protein interactions rather than as definitive evidence of biological activity.

### 2.11 Molecular dynamics simulation of drug–target complexes

Molecular dynamics (MD) simulations were performed on the docked drug–target complexes to monitor dynamic changes in their spatial conformations. Key parameters, including root mean square deviation (RMSD) and root mean square fluctuation (RMSF), were calculated to evaluate the structural stability and conformational reliability of the complexes at the molecular level.

### 2.12 Clinical safety assessment of candidate drugs

For the top candidate compounds identified through docking, adverse event reports were extracted from the FAERS database (Q1–2004 to Q2 2025). Following FDA guidelines, duplicate reports were removed, and cases with substantial missing information were excluded, retaining only those where the drug was the primary suspected agent. Adverse events were standardized and categorized using the latest MedDRA dictionary. Disproportionality analyses were conducted using ROR, PRR, BCPNN, and MGPS methods, with the reporting odds ratio method applied to eliminate potential false-positive signals. Statistical significance was set at P<0.05, enabling systematic evaluation of the clinical safety and risk profile of candidate therapeutics.

### 2.13 Cell lines and culture conditions

Four cell lines were used in this study for *in vitro* protein expression analysis and functional validation, including human normal oral keratinocytes (HOK), tongue squamous cell carcinoma cells (SCC-9), oral squamous cell carcinoma cells (CAL27), and hypopharyngeal squamous cell carcinoma cells (FaDu). HOK cells were maintained in DMEM/F12 medium, whereas SCC-9, CAL27, and FaDu cells were cultured in high-glucose DMEM medium. All culture media were supplemented with 10% fetal bovine serum (FBS) and 1% penicillin–streptomycin solution. Cells were routinely cultured and passaged in a humidified incubator at 37 °C with 5% CO_2_. Cells in the logarithmic growth phase with optimal morphology and viability were harvested for subsequent experiments.

### 2.14 Lentiviral overexpression transduction and experimental grouping

SCC-9 cells, which exhibited the most pronounced downregulation of target proteins, were selected for subsequent gene overexpression experiments. Lentiviral vectors were used to generate AIM1-overexpressing and ANXA1-overexpressing SCC-9 cell models. For functional validation of each gene, four experimental groups were established: (1) the normal control group consisting of untreated HOK cells; (2) the tumor control group consisting of untransfected SCC-9 cells; (3) the negative control group consisting of SCC-9 cells transduced with empty lentiviral vectors to control for potential effects caused by viral infection; and (4) the gene overexpression group consisting of SCC-9 cells transduced with lentiviruses carrying the corresponding AIM1 or ANXA1 overexpression constructs.

For lentiviral transduction, SCC-9 cells in the logarithmic growth phase were seeded into six-well plates. When the cell confluence reached approximately 30–40%, cells were infected with the corresponding lentiviral particles at the optimal multiplicity of infection (MOI) determined by preliminary experiments. Polybrene was added to enhance transduction efficiency. After 24 h of infection, the medium was replaced with fresh complete medium, and cells were further cultured for an additional 72 h. Following confirmation that the transduction efficiency met the experimental requirements, cells were collected for total protein extraction and subsequent analyses.

### 2.15 Western blot analysis

Total proteins were extracted from cells in each group using RIPA lysis buffer supplemented with protease and phosphatase inhibitor cocktails. Protein concentrations were determined using a bicinchoninic acid (BCA) protein assay kit. Equal amounts of protein samples were separated by sodium dodecyl sulfate–polyacrylamide gel electrophoresis (SDS-PAGE) and subsequently transferred onto polyvinylidene fluoride (PVDF) membranes using a wet-transfer system.

The membranes were blocked with 5% skimmed milk at room temperature for 1 h and then incubated overnight at 4 °C with the corresponding primary antibodies. All primary antibodies used in this study were purchased from San Ying Biotechnology, with the following dilution ratios: AIM1 (1:1000), ANXA1 (1:1000), phosphorylated NF-κB (p-NF-κB) (1:1000), total NF-κB (1:1000), and β-actin (1:20,000). After primary antibody incubation, membranes were washed three times with TBST buffer (10 min each) and incubated with horseradish peroxidase (HRP)-conjugated secondary antibodies at room temperature for 1 h.

After additional washing steps, protein bands were visualized using an enhanced chemiluminescence (ECL) detection system, and images were captured. Band intensities were quantified using ImageJ software. The relative expression levels of target proteins were normalized to β-actin, while phosphorylated NF-κB levels were further normalized to total NF-κB expression for correction of phosphorylation status.

## 3 RESULTS

### 3.1 Causal associations of immune cell type-specific eGenes with head and neck cancer

The workflow of the present study is illustrated in the graphical abstract. Using scRNA-seq-based sc-eQTL data from the OneK1K dataset and large-scale HNC GWAS summary statistics, two-sample MR analyses were performed to evaluate the causal relationships between 17 immune cell type-specific eGenes and HNC susceptibility. Following FDR correction, Cochran’s Q heterogeneity test, MR-Egger intercept assessment, and additional sensitivity analyses, no significant heterogeneity or horizontal pleiotropy was observed. A total of 494 immune cell type-specific eGenes were identified as significantly associated with HNC risk (**Supplementary Table 1**), including 252 risk-associated eGenes (OR>1) whose upregulation increased HNC susceptibility, and 242 protective eGenes (OR<1) whose upregulation reduced disease risk, providing a robust causal gene set for subsequent core target selection.

### 3.2 Differential gene expression and WGCNA in head and neck cancer

**Figure 1:**
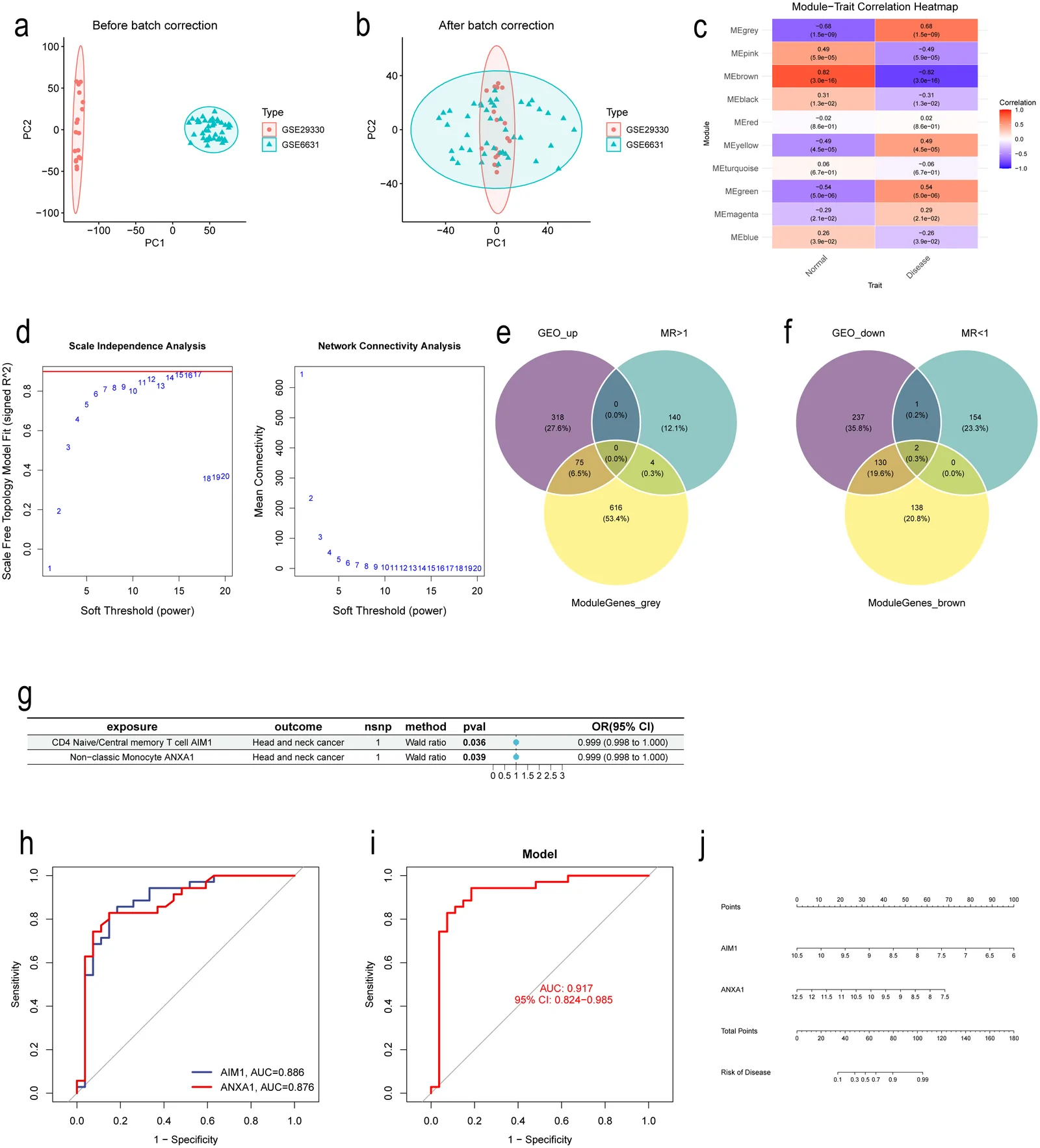
Screening of immune-related core targets in HNC, causal validation, and diagnostic performance assessment. **(a, b)** PCA before and after batch effect correction, demonstrating effective elimination of batch effects across datasets. **(c)** WGCNA module-trait correlation heatmap used to identify tumor-associated functional modules. **(d)** Selection of soft-thresholding power in WGCNA for co-expression network construction. **(e, f)** Venn diagrams depicting multi-omics intersection analysis, leading to the identification of core targets AIM1 and ANXA1. **(g)** Two-sample Mendelian randomization analysis based on sc-eQTL data, validating the genetic causal association of the identified targets with HNC. **(h)** ROC curves for individual targets, assessing the diagnostic performance of AIM1 and ANXA1 separately. **(i)** ROC curve for the combined dual-target diagnostic model, with an AUC of 0.917. **(j)** Nomogram for individualized HNC risk prediction constructed based on AIM1 and ANXA1 expression levels.

Two HNC transcriptomic datasets were retrieved from the GEO database, merged, and corrected for batch effects. Differential expression analysis was conducted with a threshold of |log_2_ fold change| ≥ 0.5, yielding 393 upregulated and 370 downregulated genes, delineating the gene expression differences between HNC and normal tissues (**Figures 1a, b**; **Supplementary Figure 1a**). Subsequently, WGCNA was performed on normalized expression data, grouping genes into 10 modules based on expression similarity. Correlation analysis between modules and HNC phenotypes identified MEgrey as the most significantly positively correlated module and MEbrown as the most significantly negatively correlated module (**Figures 1c, d**; **Supplementary Figures 1b, c**), providing a structured gene network framework for core target identification.

### 3.3 Identification of core target genes in head and neck cancer

A Venn-based intersection strategy was employed to integrate causal eGenes identified from two-sample MR analyses, differentially expressed genes from transcriptomic datasets, and WGCNA-derived core module genes, facilitating systematic identification of core HNC targets. The screening process was performed in two steps: first, the intersection of upregulated genes, positively correlated WGCNA module genes (MEgrey), and risk-associated eGenes (OR>1) was evaluated but yielded no effective targets; second, the intersection of downregulated genes, negatively correlated WGCNA module genes (MEbrown), and protective eGenes (OR<1) resulted in two overlapping genes, AIM1 and ANXA1 (**Figures 1e, f**). Forest plot analysis confirmed that both genes exhibited significant causal associations with HNC risk, thereby establishing AIM1 and ANXA1 as core target genes for HNC in this study (**Figure 1g**).

### 3.4 Construction and performance evaluation of head and neck cancer diagnostic models

Diagnostic models for HNC were constructed based on the identified core genes AIM1 and ANXA1, and the diagnostic performance of both individual genes and the combined model was systematically evaluated. ROC curve analysis revealed that each gene exhibited an AUC greater than 0.8, indicating strong independent diagnostic potential (**Figure 1h**). The combined model achieved an AUC of 0.917, demonstrating excellent overall diagnostic performance (**Figure 1i**). A nomogram was generated to quantitatively associate core gene expression levels with HNC risk, enabling visualized disease risk prediction (**Figure 1j**). Calibration curves indicated a high concordance between predicted probabilities and observed outcomes, further confirming the accuracy and clinical applicability of the AIM1- and ANXA1-based diagnostic model (**Supplementary Figure 1d**).

### 3.5 Functional characteristics of core target genes

**Figure 2:**
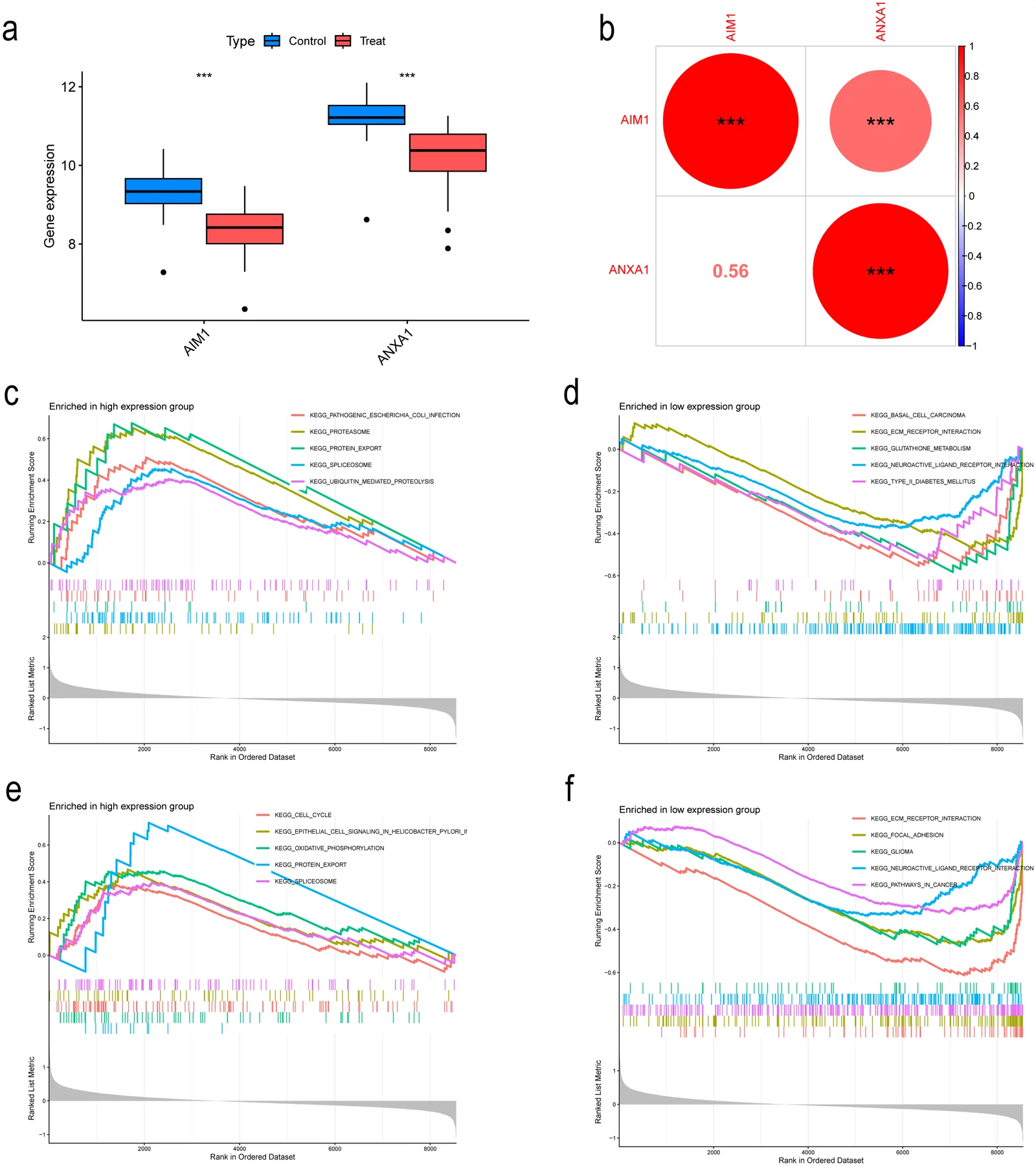
Expression validation, correlation, and functional enrichment analysis of AIM1 and ANXA1 in HNC. **(a)** Boxplots showing differential expression of AIM1 and ANXA1 between control and tumor samples, with significant downregulation observed in tumors. **(b)** Positive correlation between AIM1 and ANXA1 expression levels. **(c)** GSEA enrichment of AIM1 high-expression group in tumor proliferation-related pathways. **(d)** GSEA enrichment of AIM1 low-expression group in tumor microenvironment-associated pathways. **(e)** GSEA enrichment of ANXA1 high-expression group in tumor proliferation-related pathways. **(f)** GSEA enrichment of ANXA1 low-expression group in immune- and microenvironment-associated pathways.

Expression profiling revealed that AIM1 and ANXA1 were significantly downregulated in HNC tissues (**Figure 2a**), and their expression levels were positively correlated (**Figure 2b**). Single-gene gene set enrichment analysis indicated that high AIM1 expression was predominantly enriched in PATHOGENIC_ESCHERICHIA_COLI_INFECTION, PROTEASOME, and PROTEIN_EXPORT pathways (**Figures 2c, d**), whereas high ANXA1 expression was primarily enriched in CELL_CYCLE, OXIDATIVE_PHOSPHORYLATION, and PROTEIN_EXPORT pathways (**Figures 2e, f**). Both genes were commonly enriched in the PROTEIN_EXPORT pathway, suggesting that it may serve as a shared mechanism through which AIM1 and ANXA1 regulate HNC, while their respective pathway-specific enrichments may mediate differential effects on disease progression. GSEA revealed enrichment of several immune-related pathways in the AIM1 high-expression group, including the KEGG E. coli infection pathway. Given that this pathway contains multiple genes involved in inflammatory signaling and host immune responses, the observed enrichment may reflect shared immune-related transcriptional programs rather than a direct association with bacterial infection.

### 3.6 Association of core target genes with tumor immune infiltration

Single-sample GSEA was employed to quantitatively assess the infiltration levels of 28 immune cell subsets. The analysis revealed marked remodeling of the immune microenvironment in HNC tissues: compared to normal tissues, Gamma. Delta T cells, Type 2 T helper cells, and Regulatory T cells were significantly increased, whereas Eosinophils, Memory B cells, and Effector memory CD8 T cells were significantly decreased. Correlation analyses demonstrated specific associations between core gene expression and immune infiltration: ANXA1 was negatively correlated with Macrophages and T follicular helper cells, and positively correlated with Type 2 T helper cells; AIM1 was positively correlated with CD56^bright natural killer cells, Immature dendritic cells, and Type 2 T helper cells, and negatively correlated with T follicular helper cells. These findings indicate that AIM1 and ANXA1 may contribute to the remodeling of the HNC immune microenvironment through the regulation of immune cell infiltration (**Supplementary Figures 1e, f**).

### 3.7 Single-cell level distribution of core target genes

**Figure 3:**
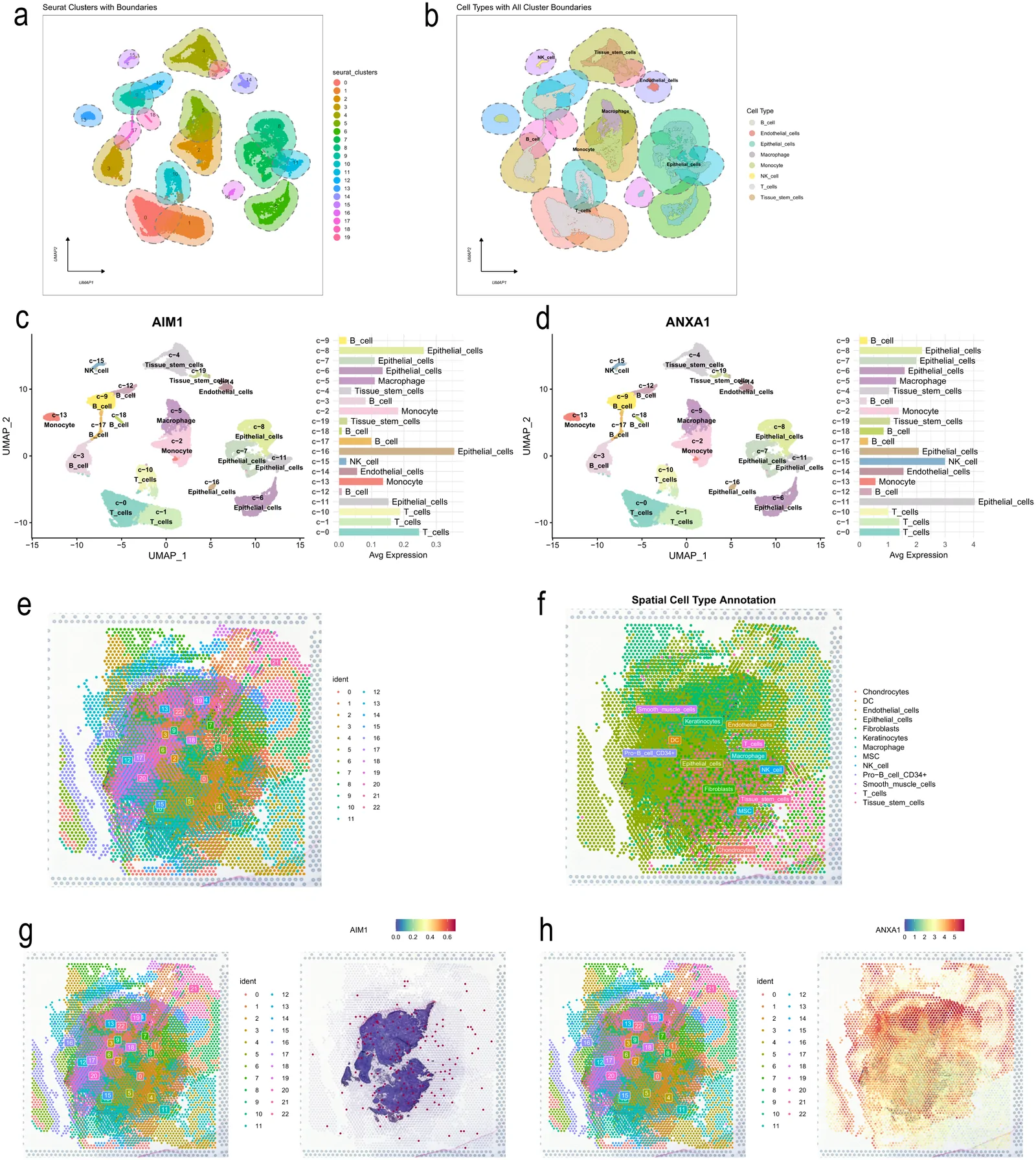
Single-cell and spatial transcriptomic analysis of core targets in HNC. **(a)** UMAP plot showing clustering of single-cell transcriptomic profiles, illustrating the segregation of HNC cell subpopulations. **(b)** UMAP plot of cell type annotation in single-cell transcriptomic data. **(c)** Expression distribution and average expression of AIM1 across single-cell subpopulations, demonstrating its immune cell-specific expression pattern. **(d)** Expression distribution and average expression of ANXA1 across single-cell subpopulations, highlighting its cell-type preference. **(e)** Spot clustering map from spatial transcriptomic data, showing spatial grouping at the tissue level. **(f)** Spatial annotation of cell types, delineating the spatial distribution of different cell populations. **(g)** Heatmap of AIM1 expression in spatial transcriptomic data, visualizing its spatial localization. **(h)** Heatmap of ANXA1 expression in spatial transcriptomic data, illustrating its spatial expression characteristics.

To elucidate the cellular origin of core target genes, the HNC scRNA-seq dataset GSE181919 from the GEO database was systematically analyzed. Following stringent quality control, normalization, and dimensionality reduction clustering, eight major cell populations were identified: B cells, Endothelial cells, Epithelial cells, Macrophages, Monocytes, NK cells, T cells, and Tissue stem cells (**Figures 3a, b**; **Supplementary Figure 1g**). Single-cell expression analysis revealed that ANXA1 was specifically enriched in Monocytes, whereas AIM1 was preferentially expressed in T cells. These findings are highly consistent with the results from sc-eQTL integrated with two-sample MR analysis, further confirming that AIM1 and ANXA1 function as immune cell type-specific regulators in HNC (**Figures 3c, d**).

### 3.8 Spatial expression patterns of core target genes

Analysis of the HNC spatial transcriptomic dataset GSE252265 identified 13 regionally distributed cell types, including Chondrocytes, Dendritic cells (DCs), Endothelial cells, NK cells, and T cells, exhibiting significant differences in spatial localization within the tumor tissue (**Figures 3e, f**). Spatial visualization of core genes AIM1 and ANXA1 demonstrated pronounced regional specificity in expression within HNC tissues, with expression patterns partially overlapping with immune cell distribution. This suggests that AIM1 and ANXA1 may exert regulatory effects closely linked to the spatial organization of the tumor immune microenvironment (**Figures 3g, h**).

### 3.9 Bayesian colocalization analysis of core target genes

**Figure 4:**
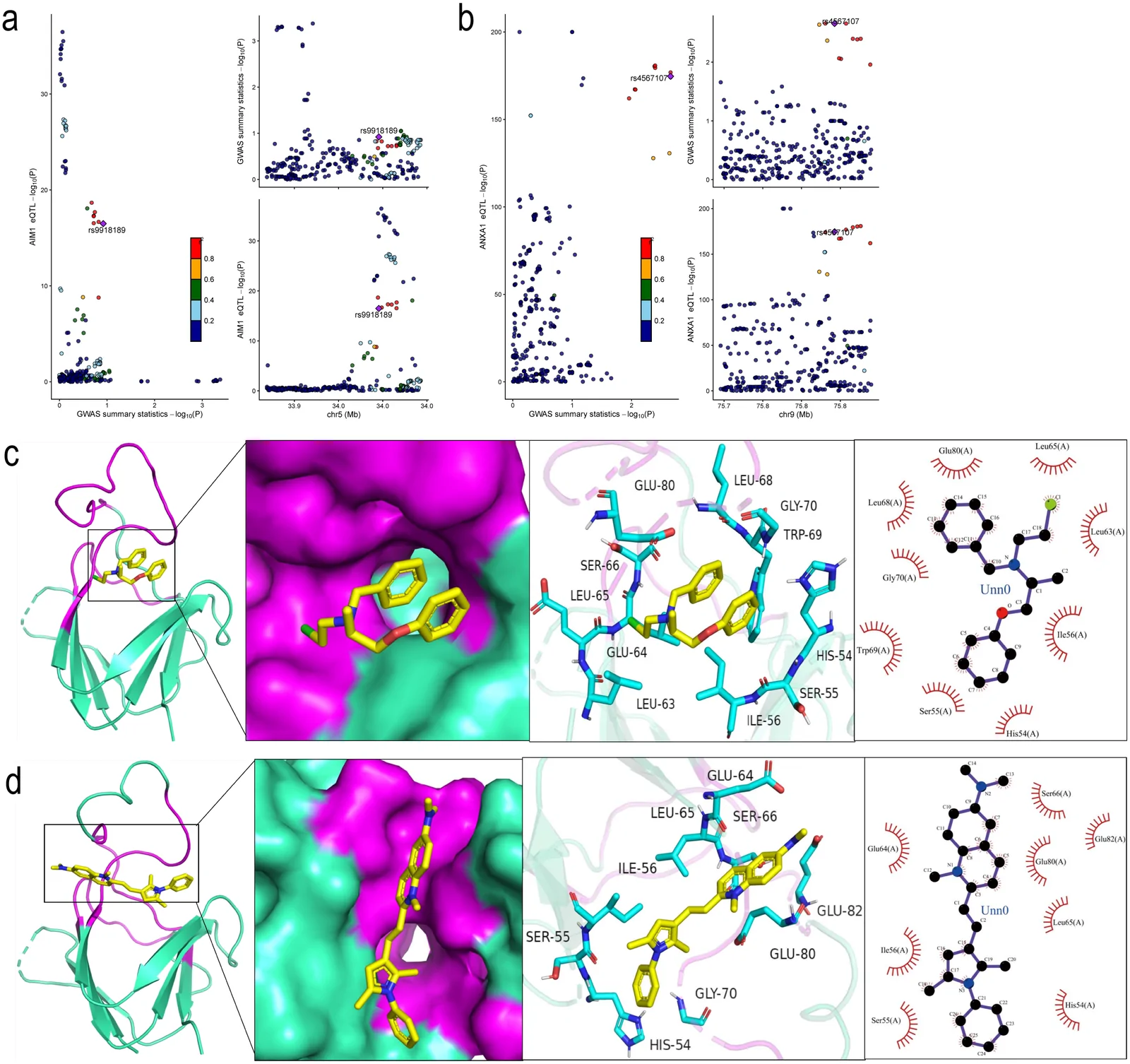
Genetic colocalization validation of AIM1 and ANXA1 and molecular docking analysis of AIM1-targeted compounds. **(a)** Colocalization analysis of AIM1, confirming its genetic causal association with HNC. **(b)** Colocalization analysis of ANXA1, validating its genetic causal association with HNC. **(c)** Molecular docking of AIM1 with phenoxybenzamine. **(d)** Molecular docking of AIM1 with pyrvinium.

To validate the causal association of core target genes with HNC at the genetic level, Bayesian colocalization analysis was performed for AIM1 and ANXA1 loci by integrating gene-specific eQTL data with HNC GWAS summary statistics. Posterior probabilities (PP) were calculated across five mutually exclusive genetic association hypotheses. The analysis identified rs9918189 as the shared causal variant for AIM1 and HNC, and rs4567107 as the shared causal variant for ANXA1 and HNC. These findings provide direct genetic evidence that AIM1 and ANXA1 loci harbor shared causal variants influencing HNC susceptibility, further confirming their validity as core HNC target genes (**Figures 4a, b**).

### 3.10 Identification of potential therapeutic compounds targeting core gene

AIM1 and ANXA1 were used as targets to query the DSigDB database for potential therapeutic compounds. Following pre-defined selection criteria, compounds with high toxicity, carcinogenicity, or teratogenicity were excluded, and only FDA-approved drugs were retained as candidates. The screening yielded three candidate drugs targeting AIM1 (topotecan, phenoxybenzamine, and pyrvinium) and three targeting ANXA1 (alpha-ergocryptine, salbutamol, and terbutaline).

### 3.10 Molecular docking validation of candidate drugs with core target genes

**Figure 5:**
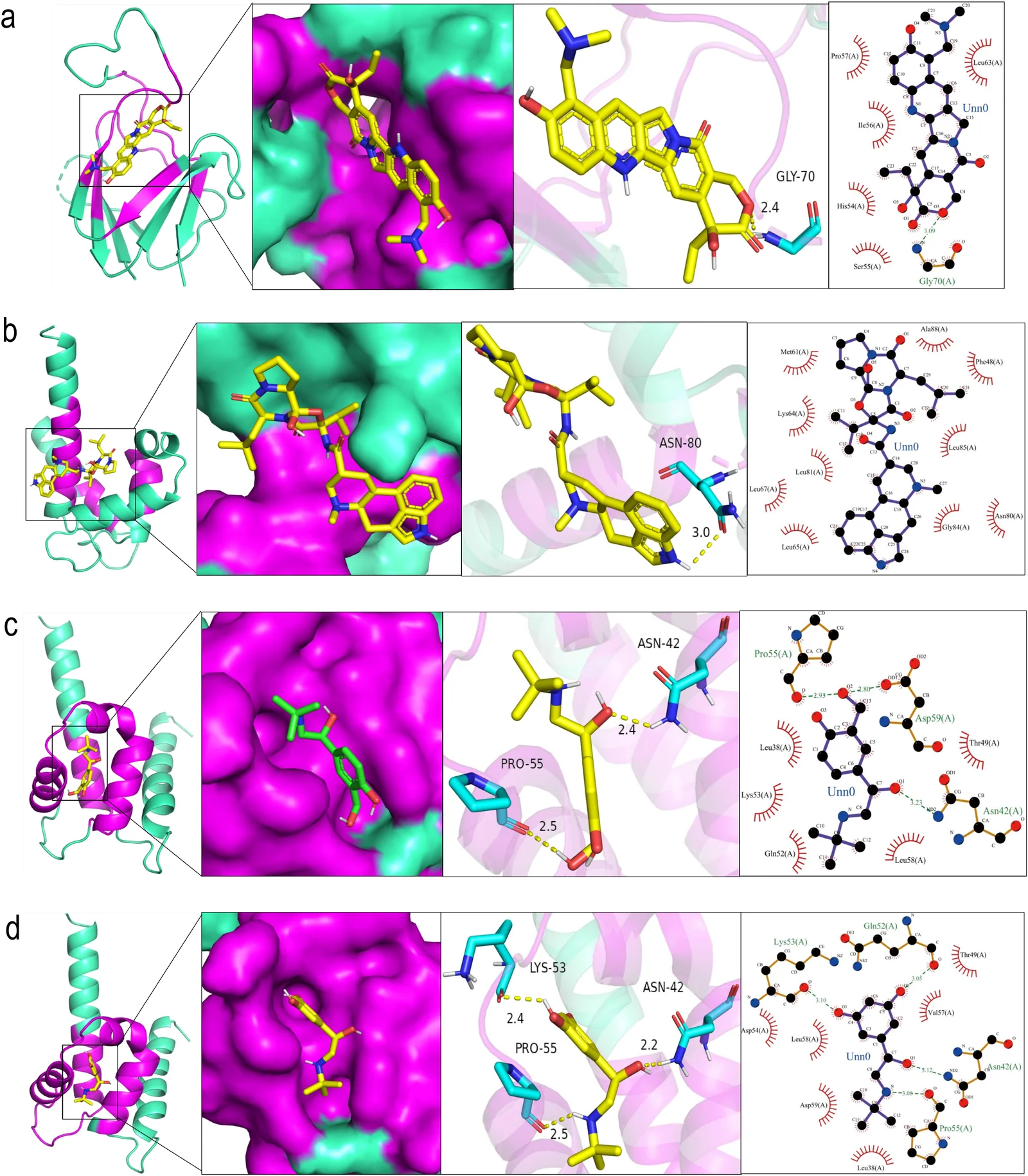
Molecular docking validation of AIM1, ANXA1, and candidate therapeutic compounds. **(a)** Molecular docking of AIM1 with topotecan, demonstrating high-affinity binding to AIM1. **(b)** Molecular docking of ANXA1 with alpha-ergocryptine, illustrating a stable binding mode with ANXA1. **(c)** Molecular docking of ANXA1 with salbutamol, confirming binding activity to ANXA1. **(d)** Molecular docking of ANXA1 with terbutaline, validating terbutaline as a high-affinity candidate targeting ANXA1.

To evaluate the binding potential of candidate drugs to proteins encoded by the core target genes, molecular docking analyses were performed. Binding sites and binding free energies were quantitatively assessed. The results demonstrated that the docking energies of AIM1 with its candidate drugs and ANXA1 with its candidate drugs were all below -5 kcal/mol, meeting the criterion for effective small molecule-protein interactions and indicating strong binding affinity (**Figures 4c, d**; **Figures 5a-d**), with detailed binding energies listed in **Supplementary Table 2**. Specifically, topotecan exhibited the lowest binding energy with AIM1 (-6.8 kcal/mol), and terbutaline exhibited the lowest binding energy with ANXA1 (-5.3 kcal/mol). These findings suggest that topotecan and terbutaline may serve as potential drug-repurposing candidates for further experimental evaluation in HNC.

### 3.11 Molecular dynamics simulation of drug–target complexes

**Figure 6:**
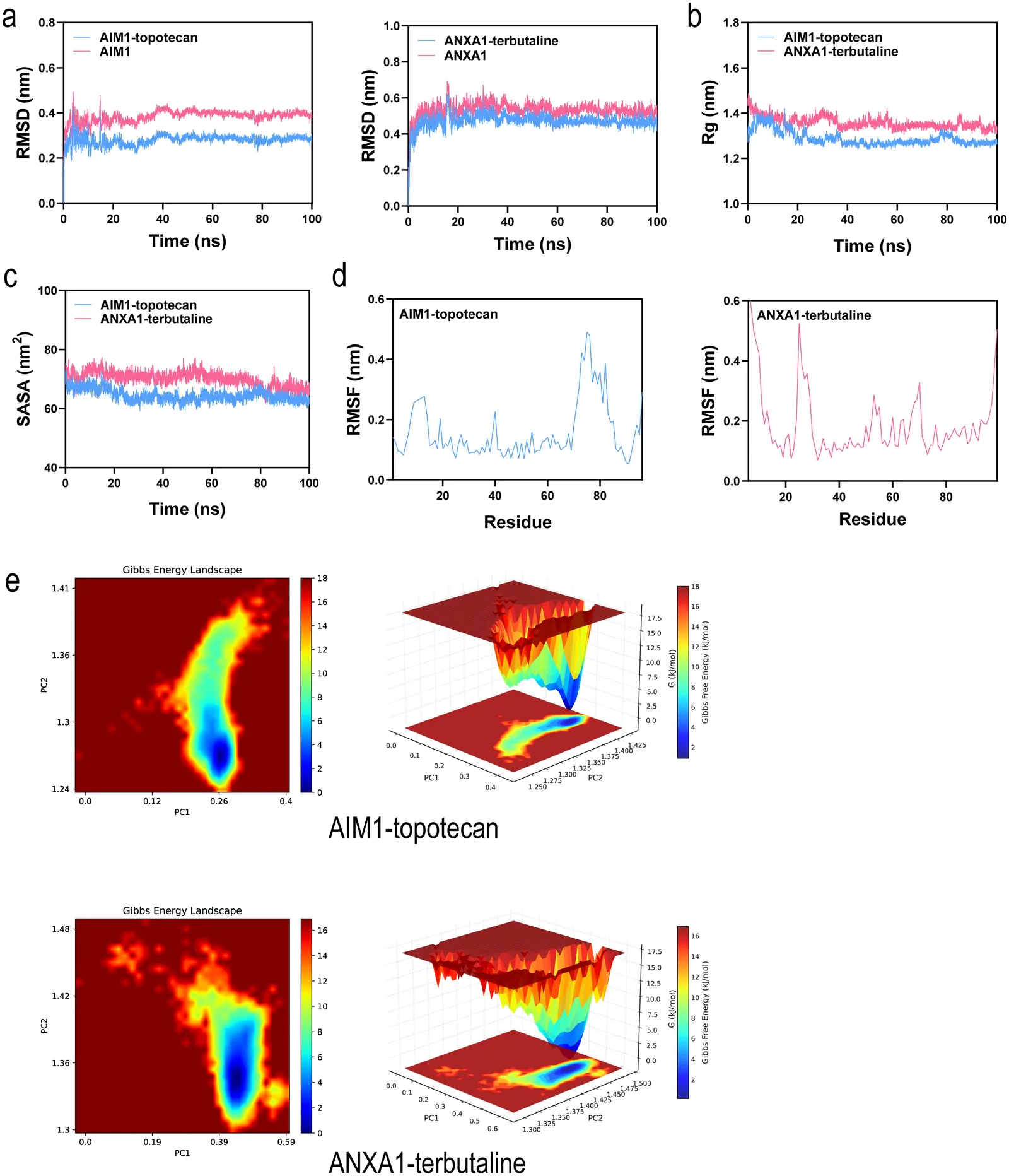
Molecular dynamics simulation analysis of protein–ligand complexes. **(a)** Time-dependent RMSD profiles of the protein–ligand complexes during molecular dynamics simulations; **(b)** Time-dependent Rg values of the protein–ligand complexes; **(c)** Time-dependent SASA changes of the protein–ligand complexes; **(d)** RMSF profiles of the protein–ligand complexes; **(e)** FEL analysis of the protein–ligand complexes.

To assess the stability of the interaction between topotecan and AIM1, a 100 ns molecular dynamics (MD) simulation was performed for the topotecan–AIM1 complex. Root mean square deviation (RMSD) is widely used to evaluate the conformational stability of protein–ligand complexes by quantifying the average deviation of atomic positions relative to the initial structure. Generally, a lower RMSD value indicates greater structural stability. Therefore, RMSD analysis was performed to assess the equilibrium and stability of the simulated systems. As shown in **Figure 6a**, both the AIM1 protein and AIM1-topotecan complex reached equilibrium after approximately 40 ns, with the RMSD values fluctuating around 0.40 nm and 0.30 nm, respectively. Similarly, the ANXA1 protein and ANXA1-terbutaline complex achieved equilibrium after approximately 20 ns, with final RMSD fluctuations of approximately 0.52 nm and 0.45 nm, respectively. These results indicate that the binding of topotecan to AIM1 and terbutaline to ANXA1 resulted in relatively stable protein–ligand complexes during molecular dynamics simulations. The radius of gyration (Rg) was calculated to evaluate the overall compactness and structural integrity of the protein–ligand complexes. As shown in **Figure 6b**, both AIM1-topotecan and ANXA1-terbutaline complexes exhibited only minor fluctuations in Rg values throughout the simulation period, suggesting that ligand binding induced limited conformational rearrangement while maintaining the overall structural compactness of the complexes. Solvent accessible surface area (SASA) analysis was subsequently performed to characterize changes in the solvent exposure of the protein–ligand interfaces (**Figure 6c**). The AIM1-topotecan and ANXA1-terbutaline complexes showed slight variations in SASA values during the simulation, indicating that ligand binding influenced the local solvent-accessible environment and resulted in moderate alterations in surface accessibility. Root mean square fluctuation (RMSF) analysis was used to investigate the flexibility of individual amino acid residues within the complexes. As presented in **Figure 6d**, the majority of residues in the AIM1-topotecan complex exhibited RMSF values below 0.50 nm, while most residues in the ANXA1-terbutaline complex showed RMSF values below 0.57 nm. These relatively low fluctuations suggest restricted residue mobility and enhanced structural stability following ligand binding. The free energy landscape (FEL) analysis was further performed to identify the dominant conformational states of the complexes (**Figure 6e**). Different colors represent distinct energy states, with red regions corresponding to higher-energy conformations and blue regions representing lower-energy and more stable conformational states. The AIM1-topotecan complex exhibited a low-energy basin at PC1 = 0.28 and PC2 = 1.40, whereas the ANXA1-terbutaline complex showed a stable energy minimum at PC1 = 0.46 and PC2 = 1.45. These low-energy regions represent the predominant stable conformations adopted by the complexes during the simulation trajectory.

**Figure 7:**
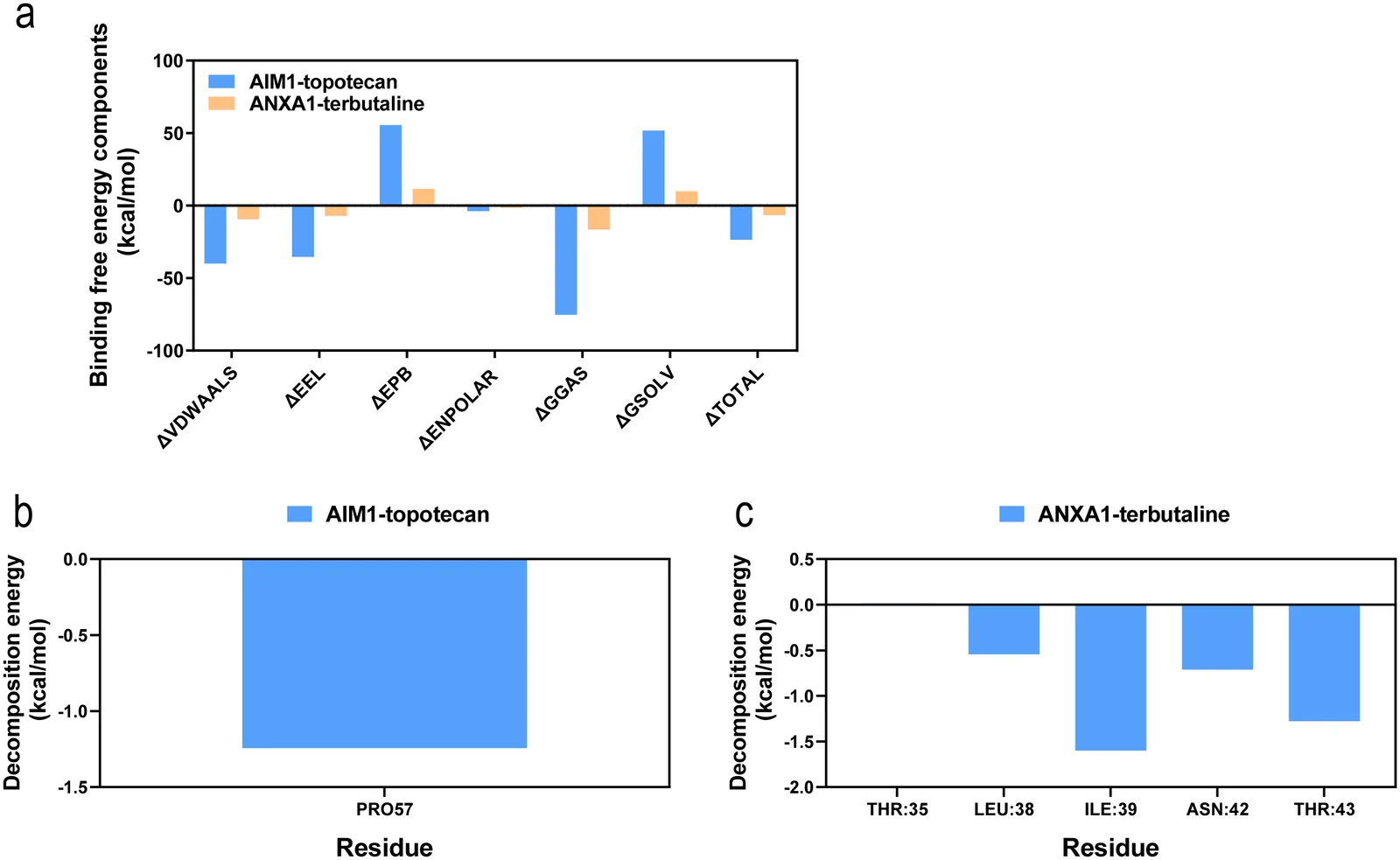
MM/PBSA-based binding free energy analysis of protein–ligand complexes. **(a)** Energy component decomposition of the protein–ligand binding free energy; **(b, c)** Per-residue decomposition analysis showing the contribution of key amino acid residues to ligand binding within the protein–ligand complexes.

Furthermore, MM/PBSA calculations were performed based on the equilibrated conformations obtained from molecular dynamics simulations to estimate the binding free energy between ligands and target proteins (**Figure 7a**). The calculated binding free energy of the AIM1-topotecan complex was −57.79 kcal/mol, indicating a favorable interaction between topotecan and AIM1. Negative binding free energy values generally suggest spontaneous and energetically favorable ligand–protein association, with more negative values reflecting stronger predicted binding affinity. To further identify key residues contributing to ligand binding, per-residue energy decomposition analysis was conducted. In the AIM1-topotecan complex, residue PRO57 exhibited a prominent contribution to ligand binding (**Figure 7b**). In the ANXA1-terbutaline complex, residues ILE39, THR43, ASN42, and LEU38 showed relatively higher energetic contributions (**Figure 7c**). These residues may participate in maintaining ligand recognition and stabilizing the protein–ligand interaction interface.

Collectively, molecular dynamics simulations demonstrated that both AIM1-topotecan and ANXA1-terbutaline complexes maintained stable conformations throughout the simulation period, supporting the favorable binding interactions between the identified small molecules and their corresponding target proteins.

### 3.12 Clinical safety assessment of candidate drugs

**Figure 8:**
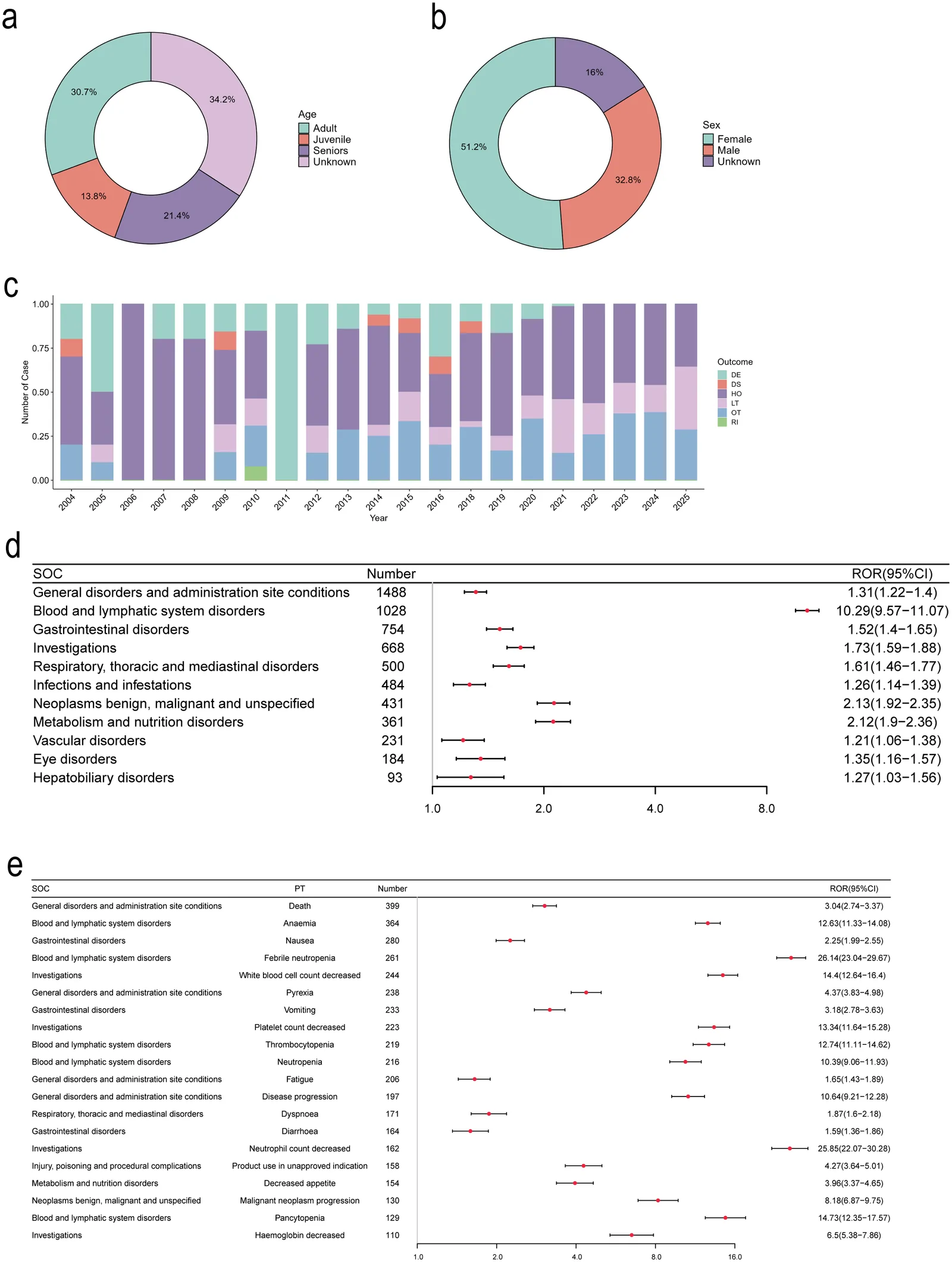
Clinical safety analysis of topotecan using the FAERS database. **(a)** Donut chart of adverse event reports by age group. **(b)** Donut chart of adverse event reports by sex. **(c)** Stacked bar chart of annual outcomes of adverse events from 2004 to 2020. **(d)** SOC forest plot, evaluating the risk of adverse events across different organ systems. **(e)** PT forest plot, highlighting specific high-risk adverse events.

To evaluate the clinical safety profile of topotecan as a potential targeted therapy for HNC, adverse event reports were extracted from the FAERS database spanning Q1–2004 to Q2 2025, where topotecan was listed as the primary suspected drug. Following deduplication and quality control, a total of 12247 reports were included. Statistical analyses indicated that topotecan-related adverse events predominantly occurred in adults, with a higher incidence in females. Clinical outcomes were primarily hospitalization (HO), prolonged hospitalization (LT), and other medical events (OT) (**Figures 8a–c**). System-level analysis revealed that adverse events mainly involved General disorders and administration site conditions, Blood and lymphatic system disorders, and Gastrointestinal disorders (**Figure 8d**), with frequently reported events including Death, Anaemia, and Nausea (**Figure 8e**).

**Figure 9:**
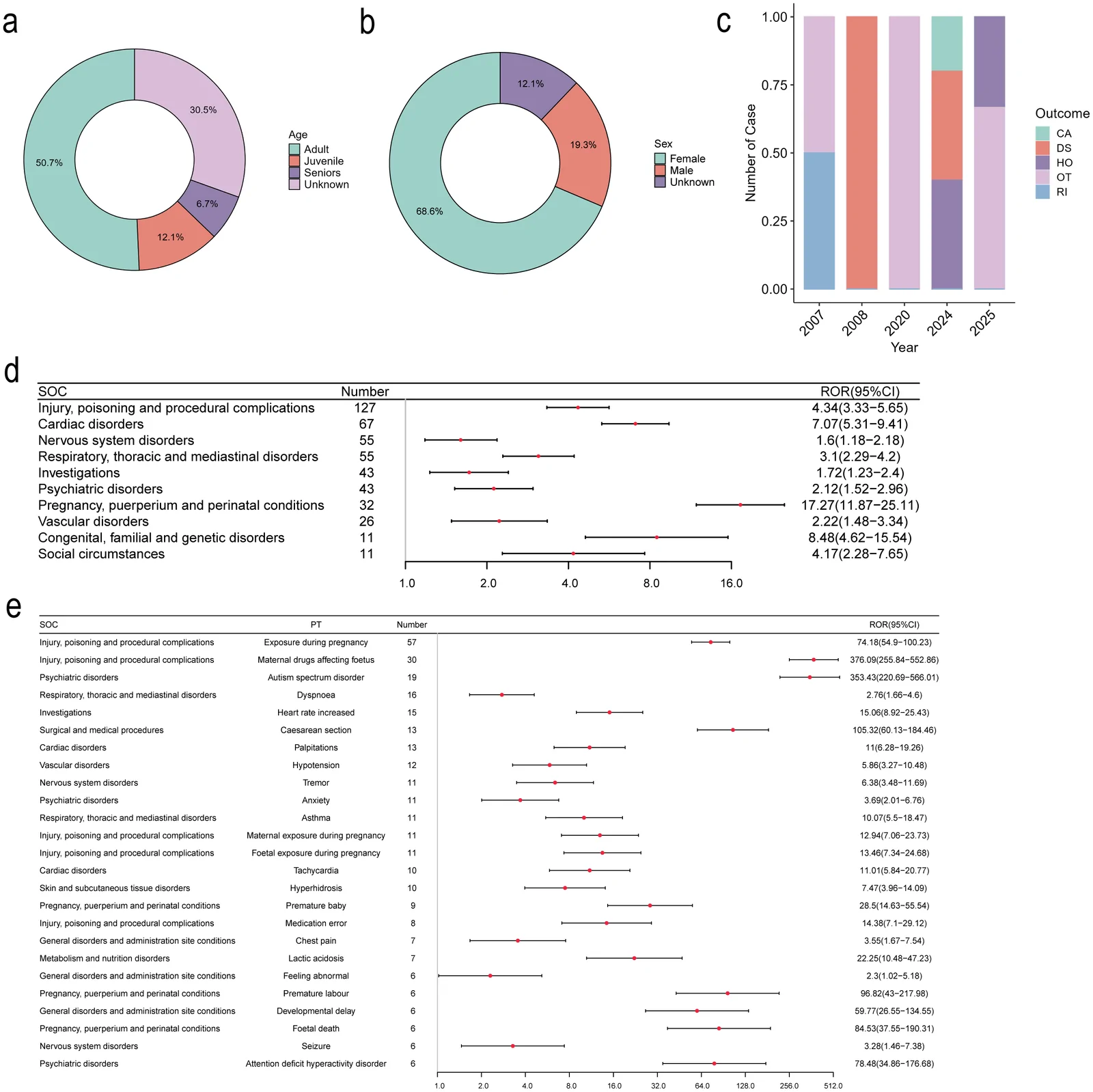
Clinical safety analysis of terbutaline using the FAERS database. **(a)** Donut chart of adverse event reports by age group. **(b)** Donut chart of adverse event reports by sex. **(c)** Stacked bar chart showing annual outcomes of adverse events. **(d)** SOC forest plot, evaluating the risk of adverse events across different organ systems. **(e)** PT forest plot, highlighting specific high-risk adverse events.

Similarly, to assess the safety of terbutaline, FAERS reports where terbutaline was the primary suspected agent were extracted, yielding 892 deduplicated and quality-controlled reports. The analysis showed that terbutaline-related adverse events primarily affected adults, predominantly females, with clinical outcomes mainly comprising hospitalization and other medical events (**Figures 9a–c**). System-level evaluation indicated major involvement of Injury, poisoning and procedural complications, and Cardiac disorders (**Figure 9d**), while frequent adverse events included Exposure during pregnancy, Maternal drugs affecting the foetus, and Autism spectrum disorder (**Figure 9e**). These signals are consistent with the established clinical application of terbutaline as a tocolytic agent and reflect its predominant use context rather than disease-specific toxicity patterns.

Taken together, these results elucidate the characteristic adverse events and high-risk factors associated with topotecan and terbutaline, providing critical guidance for their clinical translation and safe use as targeted therapies for HNC.

### 3.13 Validation of AIM1 and ANXA1 protein expression across multiple cell lines

To further validate the protein expression patterns of AIM1 and ANXA1 in head and neck cancer across multiple cellular models, the protein levels of AIM1 and ANXA1 were examined in normal oral keratinocytes (HOK) and three head and neck squamous cell carcinoma (HNSCC) cell lines derived from different anatomical sites, including SCC-9, CAL27, and FaDu.

Western blot analysis demonstrated that, compared with normal HOK cells, the protein expression levels of both AIM1 and ANXA1 were significantly reduced in all three HNSCC cell lines (SCC-9, CAL27, and FaDu), with statistically significant differences observed (all P < 0.05) (**Supplementary Figures 2a, b**). These findings were consistent with the previous transcriptomic analysis and further confirmed, at the protein level across multiple cellular models, that AIM1 and ANXA1 are consistently downregulated in HNSCC. This supports their potential roles as protective tumor suppressor candidates in head and neck cancer.

### 3.14 Regulatory effects of AIM1 and ANXA1 overexpression on NF-κB pathway activation

To investigate the potential molecular mechanisms by which AIM1 and ANXA1 regulate the progression of HNSCC, SCC-9 cells were selected to establish gene overexpression models. Western blotting was performed to assess the phosphorylation level of the core NF-κB signaling molecule p65 as an indicator of pathway activation. A total of six experimental groups were established in this study. Group A consisted of untreated normal human oral keratinocytes (HOK cells) and served as the normal control group. Group B consisted of untransfected tongue squamous cell carcinoma SCC-9 cells and served as the tumor control group. Group C consisted of SCC-9 cells transduced with an empty lentiviral vector and served as the AIM1 empty-vector negative control group to exclude nonspecific effects caused by lentiviral infection. Group D consisted of SCC-9 cells transduced with an AIM1-overexpressing lentiviral vector and served as the AIM1 overexpression group. Group E consisted of SCC-9 cells transduced with an empty lentiviral vector and served as the ANXA1 empty-vector negative control group to exclude nonspecific effects associated with lentiviral transduction. Group F consisted of SCC-9 cells transduced with an ANXA1-overexpressing lentiviral vector and served as the ANXA1 overexpression group.

For AIM1 analysis, compared with normal HOK cells in Group A, SCC-9 cells in Group B exhibited significantly reduced AIM1 protein expression (P<0.05) and markedly increased phosphorylated p65 (p-p65) protein levels (P<0.05), whereas total p65 protein expression showed no significant difference. These findings indicated aberrant activation of the NF-κB signaling pathway in HNSCC cells. Compared with Group B, cells in the AIM1 empty-vector control group (Group C) showed no significant differences in AIM1 or p-p65 protein expression (both P>0.05), confirming that lentiviral transduction alone did not cause nonspecific alterations in protein expression. Compared with Group C, AIM1 protein expression was significantly restored in Group D cells (P<0.05), accompanied by a significant reduction in p-p65 protein levels (P<0.05). Total p65 protein levels remained relatively stable among all groups, with no significant fluctuations. These results demonstrated that AIM1 overexpression inhibited p65 phosphorylation and suppressed excessive activation of the NF-κB signaling pathway in HNSCC cells (**Supplementary Figures 2c, d**). The validation experiments targeting ANXA1 showed a consistent regulatory pattern. Compared with normal HOK cells in Group A, SCC-9 cells in Group B exhibited significantly decreased ANXA1 protein expression (P<0.05) and elevated p-p65 protein levels (P<0.05). No significant differences in ANXA1 or p-p65 expression were observed between Group E (ANXA1 empty-vector control) and Group B (both P>0.05), excluding potential interference caused by lentiviral infection itself. Compared with Group E, ANXA1 protein expression was significantly restored in Group F cells (P<0.05), while p65 phosphorylation was markedly inhibited (P<0.05). Total p65 protein expression remained unchanged among all groups. These findings suggested that ANXA1 overexpression similarly suppressed aberrant activation of the NF-κB pathway in HNSCC cells by reducing p65 phosphorylation levels (**Supplementary Figures 2e, f**).

Collectively, these results indicate that both AIM1 and ANXA1 exert protective regulatory effects in HNSCC. They may participate in the regulation of malignant biological behaviors and tumor immune microenvironment remodeling in HNSCC by inhibiting phosphorylation-dependent activation of the NF-κB signaling pathway. These findings provide direct *in vitro* experimental evidence supporting the proposed tumor-suppressive regulatory mechanism of AIM1 and ANXA1 in HNSCC.

## 4 Discussion

This study leveraged sc-eQTL technology in combination with two-sample Mendelian randomization (MR) analyses to systematically identify 494 immune cell type-specific eGenes exhibiting significant causal associations with HNC. Our study does not represent the initial discovery of AIM1 or ANXA1 in cancer biology; rather, it provides a genetic and cell-type-resolved framework that prioritizes these molecules as immune-associated candidates in HNC. By integrating sc-eQTL-based genetic evidence with multi-omics validation, our work extends previous observations and offers additional insights into their potential roles within the HNC immune microenvironment. A diagnostic model based on these dual targets achieved an AUC of 0.917, reflecting their low expression in tumor tissues and specific regulatory associations with tumor immune infiltration. Further analyses revealed that AIM1 is specifically enriched in T cells, whereas ANXA1 is preferentially expressed in Monocytes, both exhibiting distinct spatial expression patterns and sharing causal genetic variants associated with HNC risk. Functionally, molecular docking and molecular dynamics simulations provided computational support for the structural compatibility and stability of the predicted ligand–protein complexes. However, these findings should be considered hypothesis-generating and require experimental validation. Furthermore, analysis of FAERS data elucidated the clinical adverse event profiles of both candidate drugs, providing critical guidance for their translational application and safe use in HNC therapy. Collectively, this study establishes the first sc-eQTL-guided framework for identifying immune-related core targets in HNC, offering novel molecular targets and experimental evidence for precision immunodiagnosis and targeted therapeutic development.

The development and progression of HNC are closely linked to the aberrant remodeling of the tumor immune microenvironment, with dysregulated expression of immune cell type-specific genes representing a central molecular driver of this process. Therefore, the precise identification of immune cell type-specific causal regulatory genes is critical for elucidating the immune regulatory mechanisms of HNC and for the development of effective diagnostic and therapeutic targets (24–26). AIM1 and ANXA1, as key tumor-suppressive genes, have been shown to regulate cell proliferation, apoptosis, inflammatory responses, and epithelial–mesenchymal transition in various malignancies, including colorectal, lung, and breast cancers. However, their specific roles and molecular mechanisms in HNC remain poorly characterized, and there is a lack of comprehensive studies integrating genetic causality with the tumor immune microenvironment (27–31). In the present study, AIM1 and ANXA1 were identified for the first time as protective, immune-related core targets in HNC from a genetic causality perspective, addressing a critical gap in HNC tumor-suppressive target research. Further analyses revealed that these genes are positively correlated in tumor tissues, share the PROTEIN_EXPORT core regulatory pathway, and harbor shared causal genetic variants associated with HNC risk, suggesting the existence of an AIM1–ANXA1-mediated cooperative tumor-suppressive regulatory network. Loss of expression or genetic variation in these genes may represent key molecular events driving HNC development. Moreover, this study is the first to integrate the tumor-suppressive functions of AIM1 and ANXA1 with the remodeling of the tumor immune microenvironment, highlighting their central role as immune regulatory targets: AIM1 is specifically enriched in T cells, whereas ANXA1 is preferentially expressed in Monocytes. Through differential regulation of distinct immune cell subset infiltration, these genes collectively contribute to the abnormal remodeling of the HNC immune microenvironment. This finding not only enriches the understanding of the molecular mechanisms underlying immune regulation in HNC but also provides a novel perspective on tumor immune evasion and establishes a theoretical foundation for further exploration of HNC immune microenvironment regulatory networks.

Insufficient early diagnosis and low specificity of existing biomarkers are major clinical challenges contributing to poor prognosis in patients with HNC. Currently available HNC diagnostic markers are largely tissue-based associative indicators, reflecting only superficial gene-disease correlations without accurately capturing the tumor immune status. Moreover, the abnormal expression of these markers is often a secondary consequence of disease progression rather than representing core molecular events driving tumor development, resulting in limited diagnostic specificity and reliability, and insufficient applicability for early precision detection. Ideal tumor biomarkers should integrate genetic causality, disease specificity, and functional relevance. AIM1 and ANXA1, identified in this study, meet these criteria comprehensively: both genes exhibit direct genetic causal associations with HNC progression, and their dysregulated expression represents core molecular events driving disease onset rather than secondary alterations. They are specifically expressed in defined immune cell populations and show pronounced downregulation in tumor tissues, demonstrating clear disease-specific expression. Additionally, AIM1 and ANXA1 are closely linked to the tumor immune microenvironment, with expression levels reflecting the degree of immune cell infiltration. Compared with conventional associative markers, these genes offer superior diagnostic specificity and reliability. Furthermore, a dual-target diagnostic model based on AIM1 and ANXA1 effectively addresses the limitations of single-gene assays, mitigating the influence of individual gene expression variability and substantially enhancing detection stability and accuracy. The combined model achieved a receiver operating characteristic area under the curve of 0.917, highlighting its strong clinical potential. The spatial expression specificity of these genes also provides a feasible framework for the development of non-invasive diagnostic technologies. Detection of AIM1 and ANXA1 expression changes or associated genetic variants in non-invasive samples such as saliva or blood could enable early, non-invasive screening for HNC, fundamentally addressing the current shortage of early diagnostic strategies.

Currently, the clinical management of HNC faces significant challenges, including a paucity of actionable targets and limited efficacy of existing therapies. Conventional surgery and chemoradiotherapy impose substantial physiological burdens on patients, while most available targeted drugs focus on single targets such as the epidermal growth factor receptor (EGFR), with long-term use often leading to drug resistance. Drug repurposing, leveraging existing clinical safety data, offers a cost-effective and expedited strategy to address the shortage of targeted therapeutics in oncology. In this study, topotecan (targeting AIM1) and terbutaline (targeting ANXA1), identified through core target screening, demonstrated strong binding affinity in molecular docking analyses. Molecular dynamics simulations further confirmed that both topotecan–AIM1 and terbutaline–ANXA1 complexes exhibit high conformational stability and binding affinity, forming stable hydrogen bond interactions. Key amino acid residues were identified as critical contributors to ligand binding, providing molecular-level validation of their feasibility as targeted therapeutics. Additionally, a comprehensive clinical safety assessment using the FAERS database elucidated the high-risk populations, affected systems, and frequently reported adverse events for both drugs. Specifically, topotecan-related adverse events predominantly involved systemic and hematological/lymphatic systems, whereas terbutaline-related events primarily affected injury, poisoning, and cardiovascular systems. It is important to interpret the FAERS findings for terbutaline within the context of its established clinical indications. Terbutaline has been widely used as a tocolytic agent, and therefore pregnancy-related adverse event reports represent the dominant exposure context in pharmacovigilance databases. The elevated reporting signals involving pregnancy exposure, fetal drug effects, and autism spectrum disorder-related reports should not be interpreted as evidence of terbutaline toxicity in HNC patients. Rather, these findings highlight the importance of considering indication-related bias and population-specific exposure patterns when interpreting spontaneous reporting data. Dedicated safety evaluation in oncology populations will require cancer-specific real-world evidence and prospective clinical assessment.

Although this study has made significant advances in identifying immune-related core targets and potential therapeutic compounds for HNC, several limitations remain that warrant further investigation. First, the genetic datasets utilized in this study, including immune cell-specific sc-eQTL data and HNC GWAS summary statistics, were predominantly derived from individuals of European ancestry. Although these datasets enabled the identification of genetically supported immune-related targets, the transferability of our findings to other ancestral populations, such as Asian and African populations, remains uncertain. Genetic heterogeneity across populations, including variations in allele frequencies, linkage disequilibrium structures, environmental exposures, and gene–environment interactions, may influence regulatory genetic effects and disease susceptibility patterns. These ancestry-dependent differences could potentially affect eQTL associations, colocalization signals, and Mendelian randomization estimates. Therefore, future studies incorporating multi-ancestry GWAS and population-specific single-cell eQTL datasets are warranted to validate the universality of AIM1 and ANXA1 as immune-related HNC targets and to determine whether population-specific regulatory mechanisms exist. Second, HNC represents a biologically diverse disease spectrum characterized by substantial heterogeneity in HPV status, anatomical subsite, molecular alterations, and immune composition. In this study, available transcriptomic datasets were analyzed collectively because comprehensive subtype annotations were limited across public resources. Therefore, although AIM1/CRYBG1 and ANXA1 were identified as immune-related candidates associated with overall HNC susceptibility, subtype-specific regulatory effects cannot be fully excluded. Future studies integrating large-scale cohorts with detailed HPV classification, anatomical annotation, and multi-omics profiling are required to determine whether these immune regulatory mechanisms are conserved across HNC subtypes or preferentially associated with specific tumor contexts. Third, the detailed molecular pathways through which AIM1 and ANXA1 regulate the tumor immune microenvironment remain insufficiently elucidated; the upstream and downstream interacting molecules, signaling mechanisms, and cooperative regulatory networks have not been fully characterized, limiting the development of precise target-based interventions. Molecular docking and molecular dynamics simulations provided computational support for the potential interaction between topotecan and AIM1 as well as between terbutaline and ANXA1. However, these approaches cannot independently determine binding selectivity or exclude interactions with alternative molecular targets. Given that both compounds possess established pharmacological activities beyond the predicted targets, potential off-target effects and pathway-level interactions should be carefully evaluated before clinical translation. Furthermore, FAERS-based pharmacovigilance analysis provided preliminary insights into the reported adverse event patterns associated with topotecan and terbutaline. However, these findings should be interpreted cautiously because spontaneous reporting systems are susceptible to several sources of bias, including polypharmacy, underlying comorbidities, treatment indications, differential reporting behaviors, and incomplete clinical information. Although disproportionality analyses can identify potential safety signals, they cannot establish causal relationships between drug exposure and adverse outcomes. Therefore, the observed associations require further validation through controlled clinical studies, real-world evidence analyses using longitudinal healthcare databases, and prospective pharmacovigilance monitoring before clinical translation. Fourth, although MR provides a framework for causal inference, the single-SNP instruments identified for AIM1/CRYBG1 and ANXA1 limit the ability to perform conventional pleiotropy sensitivity analyses. Therefore, the genetic evidence should be interpreted as supportive rather than definitive. Future studies using larger sc-eQTL datasets with multiple independent instruments will be valuable for further validating these associations. Although the AIM1/CRYBG1–ANXA1 model demonstrated favorable discrimination in the analyzed datasets, its performance should be interpreted cautiously. Because model development and evaluation were performed using related GEO datasets, the estimated AUC may be influenced by dataset-specific characteristics and potential overfitting. Independent external validation cohorts with standardized clinical annotation are required to determine its generalizability and clinical utility. Although single-cell and spatial transcriptomic analyses provided important evidence regarding the cellular origin and spatial localization of AIM1 and ANXA1, these approaches mainly represent validation-level evidence. The lack of paired tumor-normal single-cell datasets and longitudinal sampling limits the ability to reconstruct immune-state transitions or infer dynamic cell–cell communication networks. Future studies integrating larger multi-condition single-cell cohorts, spatial transcriptomics with enhanced cellular resolution, and experimental perturbation models will be necessary to elucidate the mechanistic roles of AIM1 and ANXA1 in immune regulation.

## 5 Conclusion

This study employed sc-eQTL technology as a core approach, integrating two-sample MR with multi-omics strategies, and complemented by single-cell RNA sequencing, spatial transcriptomics, and FAERS database analyses. Through this comprehensive approach, AIM1 and ANXA1 were identified as core immune-related target genes in HNC. Additionally, topotecan and terbutaline were screened and validated as potential therapeutic compounds targeting these genes. These findings provide novel molecular biomarkers for precision immunodiagnosis, offer candidate drugs for targeted therapy, and establish a foundation for improving clinical outcomes in HNC patients.

## Data Availability

The original contributions presented in the study are included in the article/**Supplementary Material**. Further inquiries can be directed to the corresponding author.

## Supplementary material

Supplementary Figure 1 Differential gene expression, WGCNA co-expression network, and immune infiltration analysis in HNC. **(a)** Volcano plot of differentially expressed genes, illustrating HNC versus normal tissue (red: upregulated; blue: downregulated; gray: not significant). **(b)** WGCNA clustering dendrogram before and after module merging, with original modules on top and merged modules below, optimizing the co-expression network. **(c)** Correlation heatmap between WGCNA modules, showing relationships among module eigengenes. **(d)** Calibration curve, validating the reliability of network construction. **(e)** Boxplot comparing immune cell infiltration abundance between HNC (red) and normal tissues (green). **(f)** Heatmap of correlations between AIM1/ANXA1 and immune cell infiltration, illustrating associations with core targets (red: positive; blue: negative). **(g)** Dendrogram of single-cell transcriptomic clustering, showing HNC sample clustering at multiple resolutions and revealing heterogeneity among cell subpopulations.

Supplementary Figure 2 Validation of AIM1 and ANXA1 protein expression and regulation of the NF-κB signaling pathway by their overexpression. **(a)** Western blot analysis of AIM1 and ANXA1 protein expression levels in normal human oral keratinocytes (HOK cells) and three head and neck squamous cell carcinoma cell lines (SCC-9, CAL27, and FaDu). **(b)** Quantitative analysis of the relative protein expression levels corresponding to the results shown in panel **(a)**. **(c, d)** Representative Western blot bands and densitometric quantification from the AIM1 overexpression experiments, showing the expression levels of AIM1, phosphorylated p65 (p-p65), and total p65. **(e, f)** Representative Western blot bands and densitometric quantification from the ANXA1 overexpression experiments.

Supplementary Table 1 Immune cell type-specific eGenes with significant genetic causal associations with head and neck cancer risk.

Supplementary Table 2 Detailed molecular docking binding energies of AIM1 and ANXA1 with candidate therapeutic compounds.
