# Supplementary material for: Integrative single-cell eQTL and multi-omics analyses reveal AIM1 and ANXA1 as immune-related hub genes and potential therapeutic targets in head and neck cancer
Source: Front Oncol. 2026 Aug 6;16:1912193. doi: 10.3389/fonc.2026.1912193 (PMC13489846; doi:10.3389/fonc.2026.1912193)
Supplement: Supplementary file 1 [file Table1.docx]

**Supplementary Table 2.** Detailed molecular docking binding energies of AIM1 and ANXA1 with candidate therapeutic compounds

| **Target protein** | **Drug** | **Binding energy (kcal/mol)** |
| --- | --- | --- |
| AIM1 | Phenoxybenzamine | -5.1 |
| AIM1 | Pyrvinium | -6.5 |
| AIM1 | Topotecan | -6.8 |
| ANXA1 | Alpha-ergocryptine | -5.1 |
| ANXA1 | Salbutamol | -5.1 |
| ANXA1 | Terbutaline | -5.3 |
